# Supplementary material for: Genomes of cultivated and wild Capsicum species provide insights into pepper domestication and population differentiation
Source: Nat Commun. 2023 Sep 7;14:5487. doi: 10.1038/s41467-023-41251-4 (PMC10484947; doi:10.1038/s41467-023-41251-4)
Supplement: Supplementary file 1 — Supplementary Information [file 41467_2023_41251_MOESM1_ESM.pdf]

Genomes of cultivated and wild *Capsicum* species provide insights into pepper domestication and population differentiation

Liu *et al.*

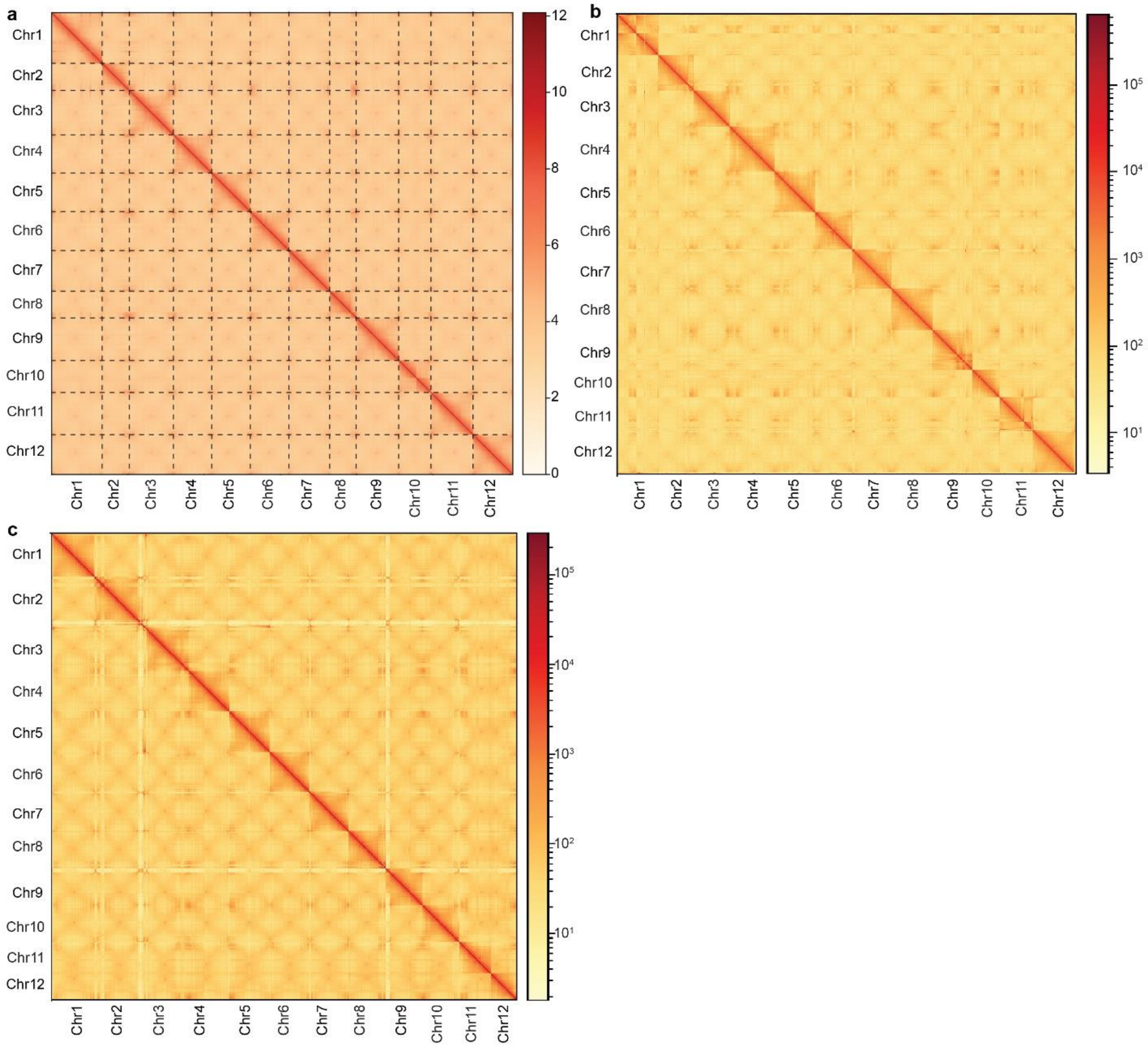

Supplementary Fig. 1. Hi-C interaction heatmap of three *Capsicum* genomes. a, Hi-C interaction heatmap of the *C. annuum* var. *annuum* Zhangshugang genome. **b**, Hi-C interaction heatmap of the *C. baccatum* var. *pendulum* PI 632928 genome. **c**, Hi-C interaction heatmap of the *C. pubescens* Grif 1614 genome.

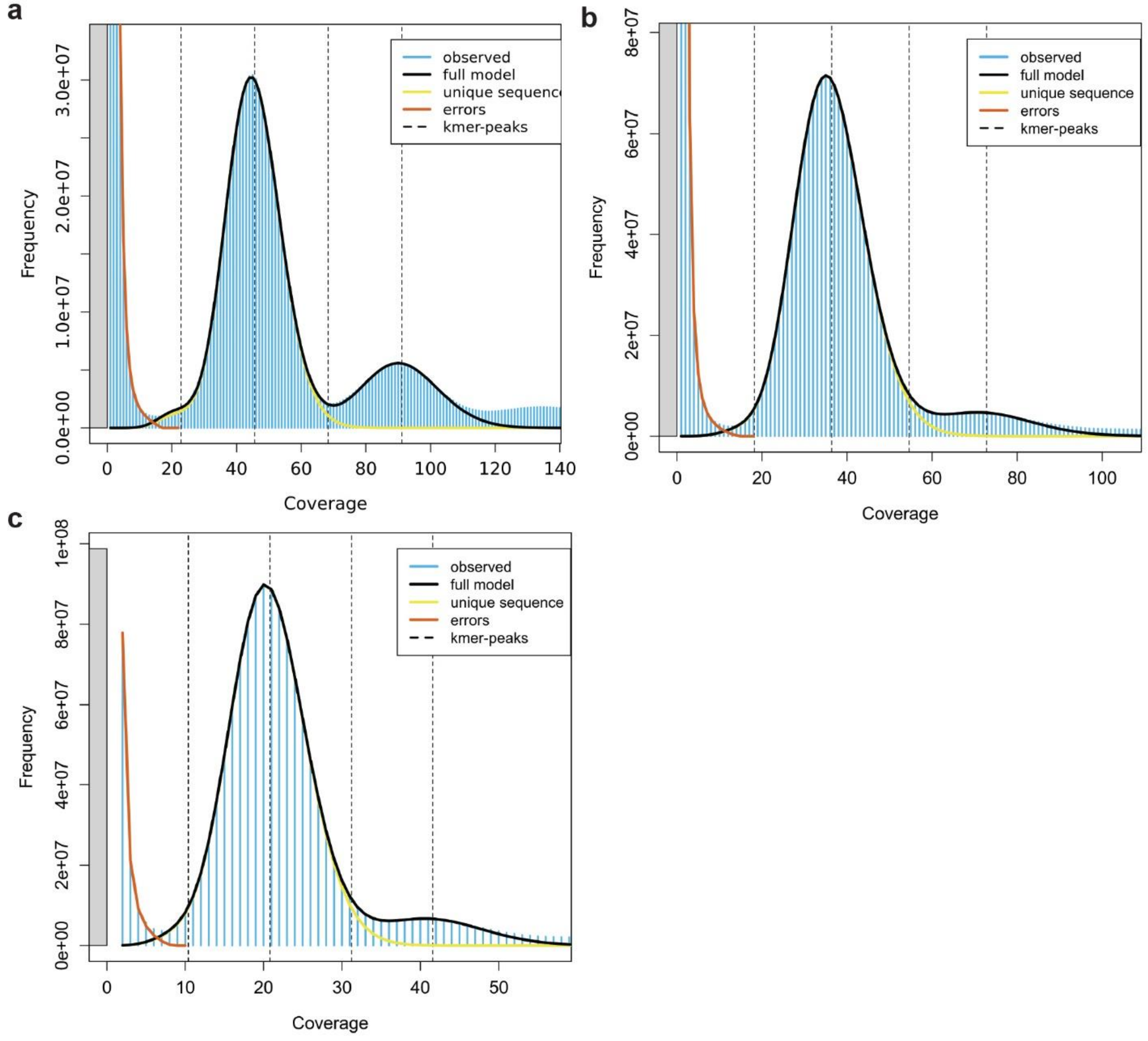

Supplementary Fig. 2. k-mer analyses of Illumina/HiFi sequencing reads of *C. annuum* var. *annuum* Zhangshugang (a), *C. baccatum* var. *pendulum* PI 632928 (b) and *C. pubescens* Grif 1614 (c), using GenomeScope (https://github.com/schatzlab/genomescope). The analyses indicated an estimated genome size of 3,058,784,992 bp and a heterozygosity level of 0.076% for Zhangshugang, 3,430,278,101 bp and 0.035% for PI 632928, and 4,158,269,623 bp and 0.051% for Grif 1614.

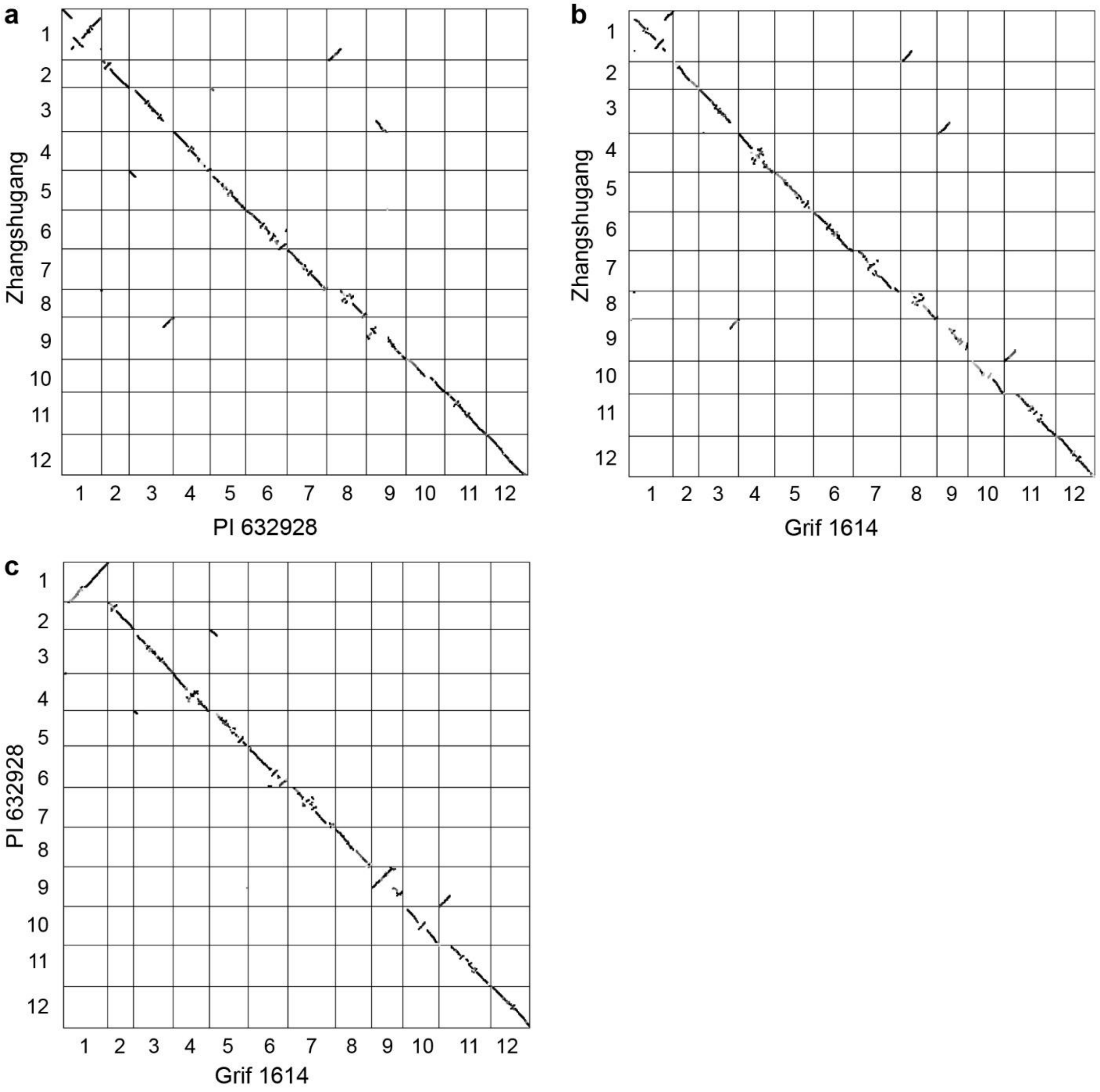

Supplementary Fig. 3. Synteny between pepper genome assemblies. a, Synteny between *C. annuum* var. *annuum* Zhangshugang and *C. baccatum* var. *pendulum* PI 632928 genome assemblies. **b**, Synteny between *C. annuum* var. *annuum* Zhangshugang and *C. pubescens* Grif 1614 genome assemblies. **c**, Synteny between *C. baccatum* var. *pendulum* PI 632928 and *C. pubescens* Grif 1614 genome assemblies.

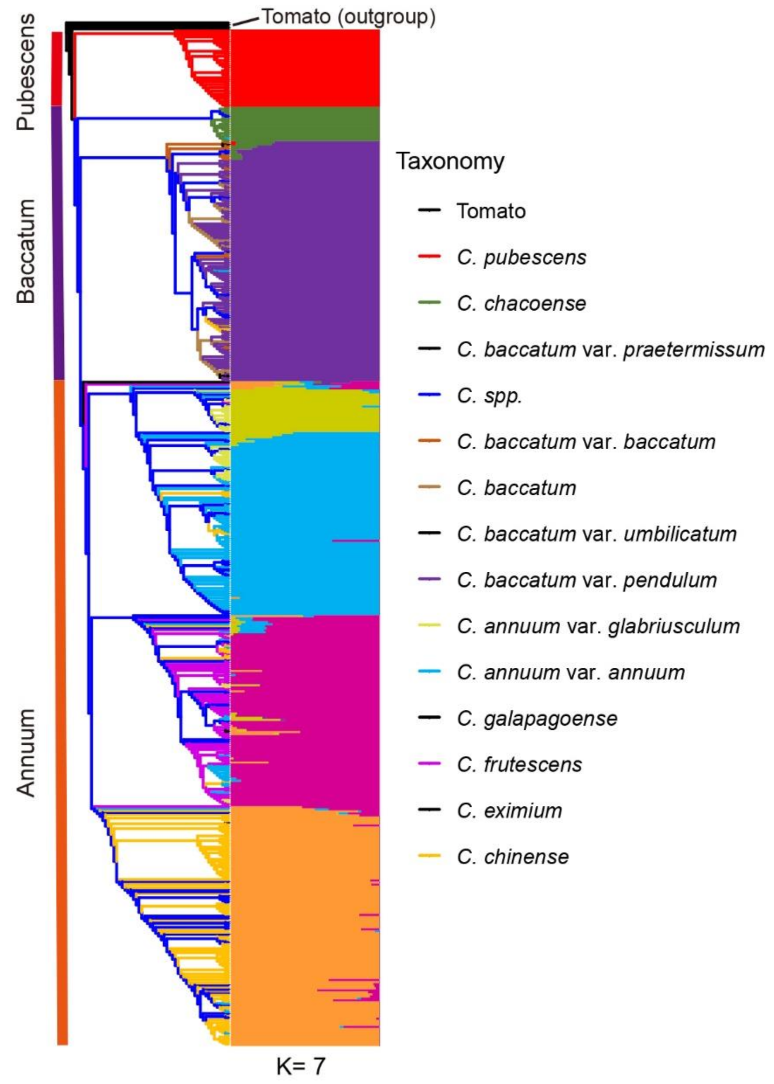

Supplementary Fig. 4. Maximum likelihood phylogenetic tree and population structure of the 500 accessions in the pepper core collection before taxonomy correction.

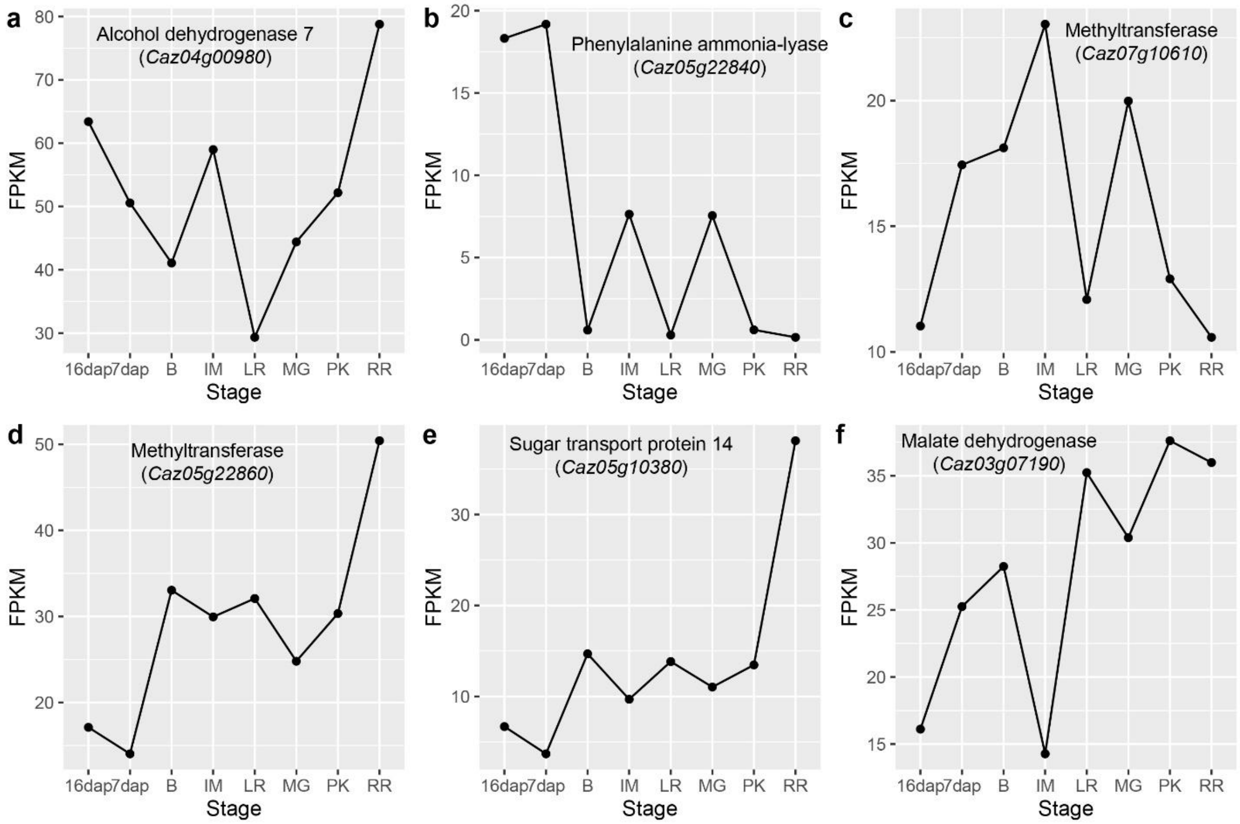

Supplementary Fig. 5. Expression profiles of fruit flavor-related genes in the highly differentiated genome regions during the fruit development of Zhangshugang. a, Expression profile of *Caz04g00980*. **b**, Expression profile of *Caz05g22840*. **c**, Expression profile of *Caz07g10610*. **d**, Expression profile of *Caz05g22860*. **e**, Expression profile of *Caz05g10380*. **f**, Expression profile of *Caz03g07190*. dap, days after pollination; IM, immature; MG, mature green; B, breaker; PK, pink; LR, light red; RR, red ripe.

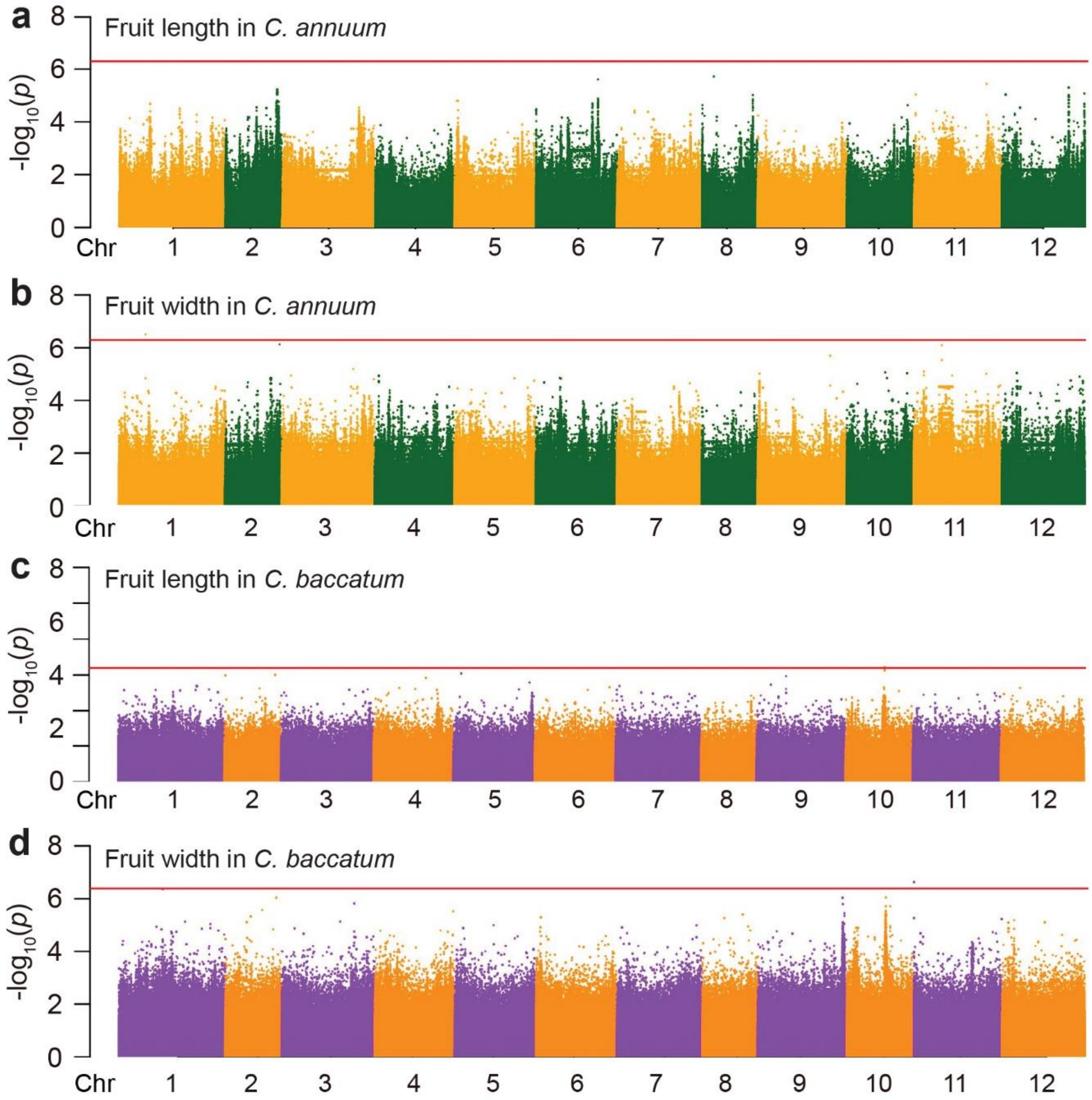

Supplementary Fig. 6. Manhattan plots of GWAS of fruit length (a,c) and fruit width (b,d) of *C. annuum* and *C. baccatum*. Red horizontal line corresponds to the genome-wide significant threshold.

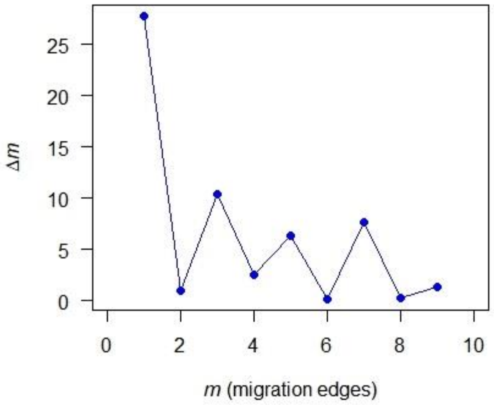

Supplementary Fig. 7. Model fits for TreeMix with migration ranging from 0 to 10, each with 10 replicates. The optimal number of migration edges is indicated by the peak of Δm (m = 1) based on the Evanno model in OptM (https://cran.r-project.org/web/packages/OptM/).

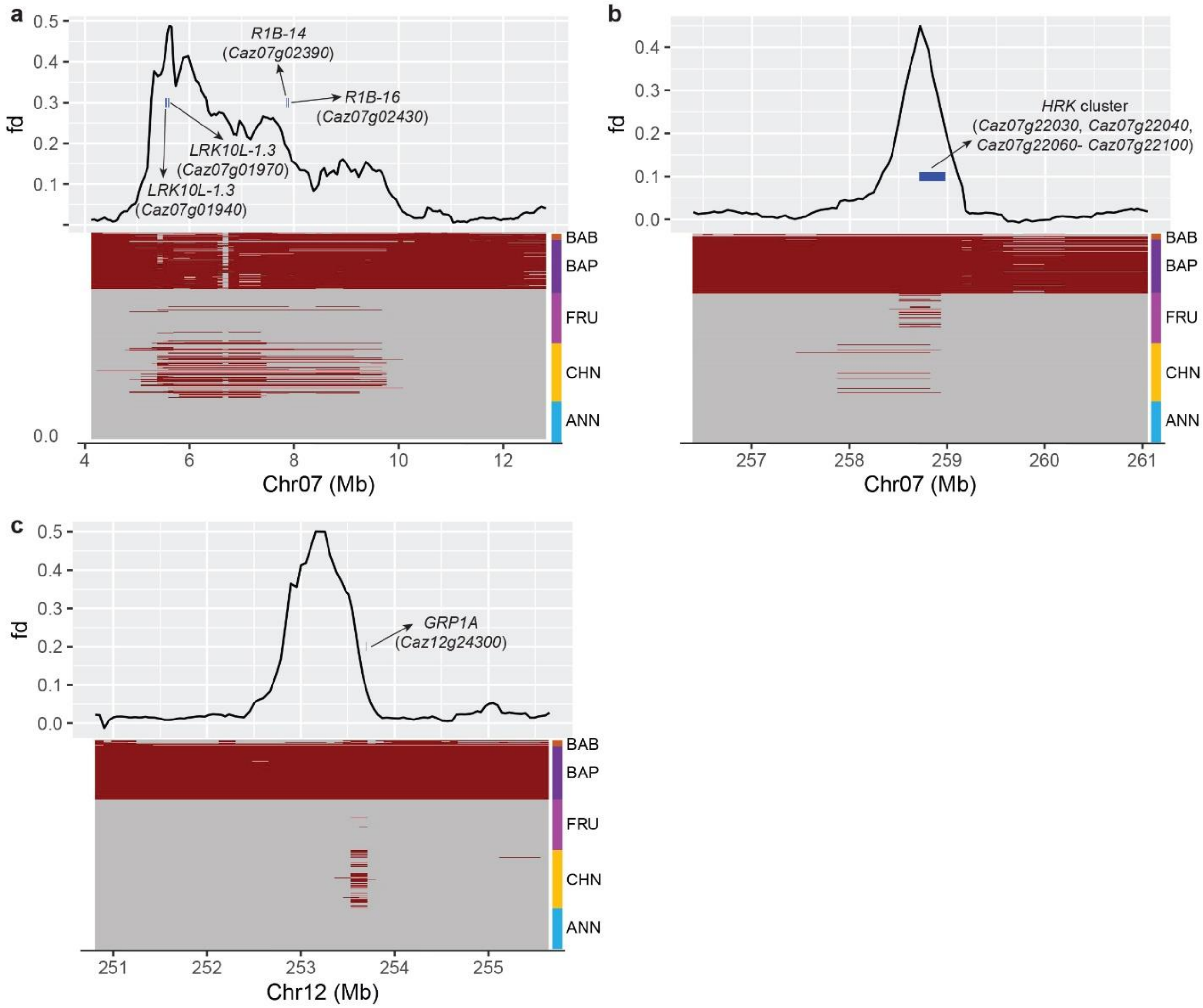

Supplementary Fig. 8. Degree of introgression (*fd*) and introgressed regions from *C. baccatum* to *C. chinense* and *C. frutescens*, at three genome regions on chromosome 7 (a and b) and 12 (c). Dark red color represents alleles dominant in *C. baccatum* accession (allele frequency ≥ 0.8). BAB, *C. baccatum* var. *baccatum*; BAP, *C. baccatum* var. *pendulum*; FRU, *C. frutescens*; CHN, *C. chinense*. *LRK10L-1.3*, Leaf Rust 10 Disease-resistance Locus Receptor-like Protein Kinase-like 1.3; *R1B-14*, Late Blight Resistance Protein Homolog R1B-14; *R1B-16*, Late Blight Resistance Protein Homolog R1B-16; *HRK*, Heat-related Receptor Kinase; *GPR1A*, Glycine-rich RNA-binding Protein GRP1A.

Supplementary Table 1. Summary statistics of sequences generated for genome assemblies.

Accession	Species	PacBio*			Illumina		Hi-C	
		No. reads	Total bases	Mean (bp)	No. reads	Total bases	No. reads	Total bases
Zhangshugang	*C. annuum* var. *annuum*	20,137,345	372,420,058,430	18,494	531,390,974	158,582,344,710	2,132,567,622	318,109,637,813
PI 632928	*C. baccatum* var. *pendulum*	13,088,981	275,775,618,948	21,069	500,120,600	150,036,180,000	1,156,596,806	346,979,041,800
Grif 1614	*C. pubescens*	5,971,418	84,857,534,240	14,210	NA	NA	832,951,291	249,885,387,300

*CLR reads were generated for Zhangshugang and PI 632928, and HiFi reads were generated for Grif 1614

Supplementary Table 2. Summary of pseudomolecules of the three assembled *Capsicum* genomes.

Accession	*C. annuum* var. *annuum* Zhangshugang		*C. baccatum* var. *pendulum* PI 632928		*C. pubescens* Grif 1614	
**Chromosome**	**No. contigs**	**Total bases**	**No. contigs**	**Total bases**	**No. contigs**	**Total bases**
Chr01	53	332,589,375	73	283,018,573	55	360,886,595
Chr02	27	177,306,215	47	190,167,000	7	206,417,740
Chr03	40	289,771,274	81	311,655,000	12	319,883,835
Chr04	26	248,920,013	87	257,282,916	20	289,728,695
Chr05	64	254,842,644	70	250,090,119	25	312,177,810
Chr06	82	253,193,053	104	286,498,000	16	320,946,106
Chr07	54	266,356,021	79	283,654,608	49	382,437,095
Chr08	30	174,311,981	85	277,193,832	18	295,017,736
Chr09	42	278,389,512	46	279,988,019	8	248,759,183
Chr10	31	210,317,287	202	272,580,773	30	290,266,911
Chr11	37	275,167,330	63	286,684,000	37	421,488,110
Chr12	36	259,718,875	195	288,445,679	18	308,455,391
Total (chromosome)	522	3,020,883,580	1,132	3,267,258,519	295	3,756,465,207
Unanchored	79	2,722,584	626	42,214,723	61	170,759,818
Total	601	3,023,606,164	1,758	3,309,473,242	356	3,927,225,025

Supplementary Table 3. Quality evaluation of the three genomes using Merqury.

Category	Zhangshugang	PI 632928	Grif 1614
k-mers uniquely found only in the assembly	7,723,354	1,891,728	25,269
k-mers found in both assembly and the read set	3,024,665,041	3,310,651,728	3,927,307,206
QV score	38.93	45.65	65.14
Error rate	0.000128	0.0000272	0.00000031
Total solid k-mers in the assembly	1,610,057,005	1,709,957,543	1,739,098,861
Total solid k-mers in reads	1,646,745,771	1,720,576,625	1,752,668,621
Completeness	97.77%	99.38%	99.23%

Supplementary Table 4. **Summary of repeat sequences in the three *Capsicum* genomes.**

TE type	Zhangshugang		PI 632928		Grif 1614	
	Length (bp)	% Genome	Length (bp)	% Genome	Length (bp)	% Genome
DNA	221,047,316	7.31	155,547,944	4.70	273,027,122	6.95
LINE	78,428,286	2.59	34,432,008	4.01	69,992,650	1.78
SINE	5,181,813	0.17	-	-	2,185,733	0.06
LTR	1,842,664,163	60.94	2,130,779,962	64.35	2,875,090,075	73.20
Satellite	3,002,013	0.10	-	-	-	-
Simple repeat	18,424,827	0.61	1,297,629	0.08	95,126	0.00
Other	4,924	0.00	2,573,047	0.04	9,676,139	2.50
Unknown	290,362,082	9.60	563,341,822	17.01	275,310,858	7.01
Total	2,363,580,107	78.16	2,887,972,412	87.21	3,505,377,703	89.25

Supplementary Table 5. Summary of SNP and small indel annotations.

Category	No. SNPs	No. small indels
Intergenic	96,285,087	4,915,149
Intron	2,835,323	315,919
5'/3' UTR	190,914	41,589
Synonymous	294,543	NA
Non-synonymous	430,414	NA
Splicing	48,100	6,343
Start gain/loss	13,396	77
Stop gain/loss	13,855	160
Inframe insertion/deletion	NA	9,179
Exon gain/loss	NA	20
Frameshift	NA	18,543
**Total**	**100,111,632**	**5,306,979**

Supplementary Table 6. Genes in the GWAS signals of fruit shape.

GWAS panel	Gene ID	Gene start	Gene end	Gene annotation
C. annuum	Caz02g22950	160,935,913	160,942,127	Ubiquitin-conjugating enzyme E2
	Caz02g22960	160,943,207	160,948,989	Folate transporter 1
	Caz02g22970	160,955,919	160,957,706	Peroxidase
	Caz02g22980	160,961,455	160,963,223	Peroxidase
	Caz02g22990	160,974,026	160,996,743	Unknown
C. baccatum	Caz10g08850	120,592,246	120,593,428	Ovate protein family
	Caz10g08860	121,805,884	121,815,984	Unknown
	Caz10g08870	122,080,120	122,082,275	YLP motif-containing protein 1-like
	Caz10g08880	122,408,199	122,415,044	Aspartate/other aminotransferase
