# Supplementary material for: Genomes of cultivated and wild Capsicum species provide insights into pepper domestication and population differentiation
Source: Nat Commun. 2023 Sep 7;14:5487. doi: 10.1038/s41467-023-41251-4 (PMC10484947; doi:10.1038/s41467-023-41251-4)
Supplement: Supplementary file 3 — Description of Additional Supplementary Files [file 41467_2023_41251_MOESM3_ESM.pdf]

Description of Additional Supplementary Files

Supplementary Data 1
Summary statistics of pepper genome assemblies

Supplementary Data 2
Summary statistics of small indels and SVs identified in PI 632928 and Grif 1614 relative to Zhangshugang

Supplementary Data 3
Summary information of the 1296 accessions from the *Capsicum* genus

Supplementary Data 4
Sequence summary of the 500 core accessions

Supplementary Data 5
Genes related flowering time, flavor and biotic/abiotic stress responses in highly differentiated genome regions among the five domesticated *Capsicum* species

Supplementary Data 6
Potential selective sweeps during the domestication of *C. annuum* var. *annuum*.

Supplementary Data 7
Putative selective sweeps during the domestication of *C. baccatum* var. *pendulum*

Supplementary Data 8
Genes/QTLs related to fruit size, shape and pungency in domestication sweeps of *C. annuum* var. *annuum* and *C. baccatum* var. *pendulum*

Supplementary Data 9
Genes in the introgressed regions from *C. baccatum* to *C. frutescens* and *C. chinense*
